# Tuberculous Dactylitis: An Uncommon Presentation of a Common Infection

**DOI:** 10.1155/2016/4013471

**Published:** 2016-01-17

**Authors:** G. Nayantara Rao, Jayasri Helen Gali, S. Narasimha Rao

**Affiliations:** ^1^Department of Pediatrics, Apollo Institute of Medical Sciences and Research, Hyderabad 500096, India; ^2^Department of Pulmonology, Apollo Institute of Medical Sciences and Research, Hyderabad 500096, India

## Abstract

Tuberculous dactylitis is an unusual form of osteoarticular tuberculosis involving the short tubular bones of hands and feet, which is uncommon beyond six years of age. We report the case of a fifteen-year-old adolescent boy who was diagnosed with tuberculous dactylitis, involving contralateral hand and foot. His diagnosis was delayed due to lack of suspicion of this rare entity. The report also examines the diagnostic difficulties faced by clinicians in arriving at an appropriate diagnosis.

## 1. Introduction

Tuberculous infection of metacarpals, metatarsals, and phalanges of hands and feet is known as tuberculous dactylitis. Eighty-five percent of the patients are younger than six years of age [1] and data relating to tuberculous dactylitis among adolescents is scarce. To the best of our knowledge, simultaneous involvement of both the limbs in a child above six years of age is extremely rare and has been hardly reported.

## 2. Case Report

A fifteen-year-old adolescent boy initially presented with a dull aching pain followed by a swelling over the dorsum of his right foot. After a fortnight, he noticed a painful swelling on the dorsum of his left hand, which gradually progressed over the last six months. Initially, he was taken to a general practitioner after a month of his presenting complaints, for which he was prescribed analgesics for pain and oral antibiotics (a combination of amoxicillin and clavulanic acid) for a period of 10 days suspecting a pyogenic infection, but to no avail. However, the swelling persisted only to increase in size, forming an abscess. The child consulted another doctor after 3 months, where both the abscesses were incised and drained leading to discharging sinuses. The pain persisted despite the treatment causing restricted movements of the fingers and toes. Finally, he was brought to our Outpatient Department, where we admitted him to our ward. On carefully probing into the history, the parents told us that the child's grandfather was diagnosed with pulmonary tuberculosis and was taking treatment for the same for 5 months. However, the child had no history of fever, cough, night sweats, loss of weight, or trauma.

Local examination revealed an oval shaped swelling of 4 cm ∗ 2 cm over the right 1st metatarsal bone with a discharging sinus and another swelling over the left 2nd metacarpal bone of 2 cm ∗ 1 cm. Both swellings were hard and fixed to the underlying bone. There was tenderness and local rise of temperature on palpation. Movements were restricted at the left proximal interphalangeal joint of the index finger and the right great toe. There was no lymphadenopathy. Systemic examination was unremarkable.

**Figure 1:**
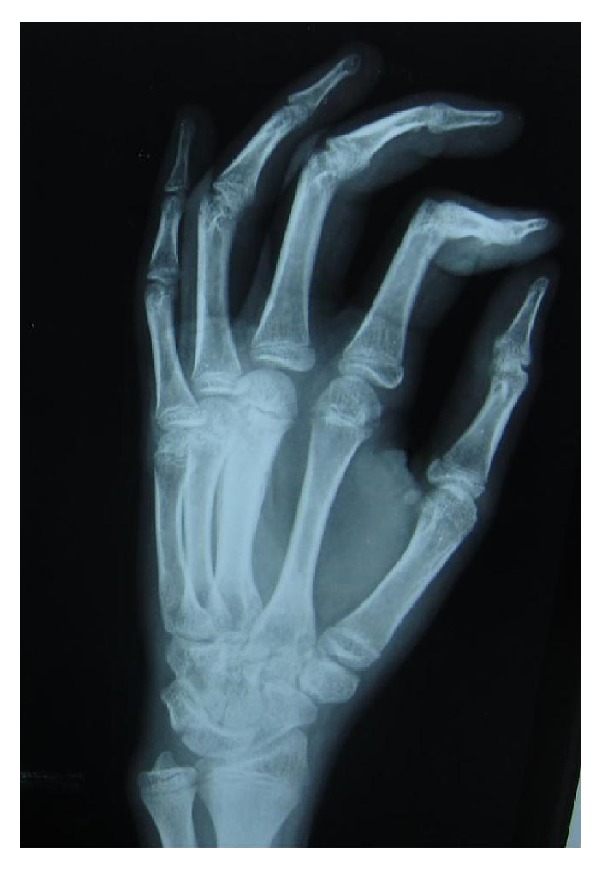
Radiograph of left hand (lateral view), showing cortical thickening and expansion of third metacarpal predominantly involving diaphysis with subperiosteal reaction.

**Figure 2:**
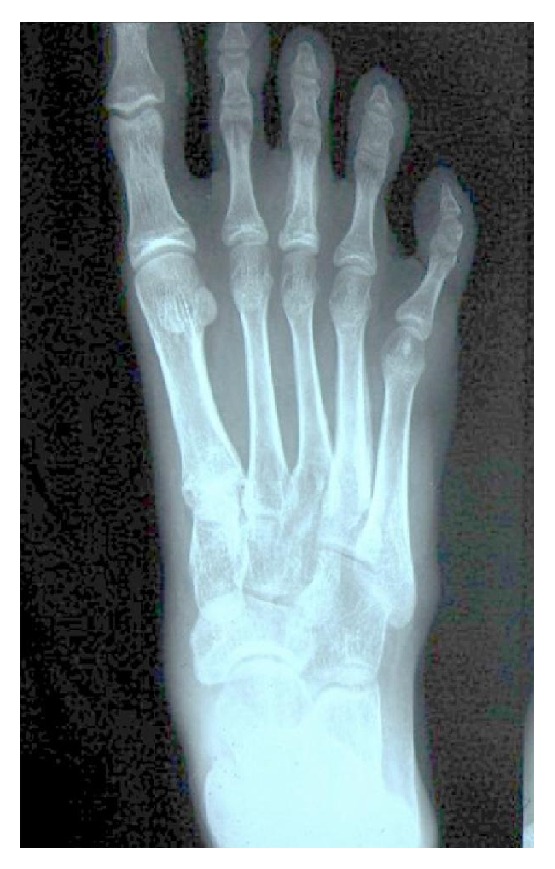
Radiograph of right foot (AP view), showing irregular destruction of proximal phalanx of 3rd toe and cortical thickening of 4th metatarsal with solid periosteal reaction.

Complete laboratory investigations were done which revealed a haemoglobin of 10.5 g/dL, total leukocyte count of 11,600/mm^3^, and an ESR of 90 mm/hr, and Mantoux test was strongly positive (20 mm). Chest radiograph was normal. Open biopsy specimen for histopathological examination taken from the 1st metatarsal of right foot revealed a caseating granulomatous inflammation consistent with tuberculosis, but staining for mycobacterium was negative. Radiograph of the left hand showed a diffuse thickening of 2nd metacarpal with subperiosteal new bone formation (Figure 1). Radiograph of the right foot showed central cystic lesion with diffuse thickening of metatarsal bone and proximal phalanx of the great toe (Figure 2). Based on all these features, a probable diagnosis of tuberculous dactylitis was established and antitubercular therapy was initiated, which consisted of a four-drug regimen (Isoniazid, Rifampicin, Pyrazinamide, and Ethambutol) for a period of two months and two-drug regimen (Isoniazid and Rifampicin) for four months as per the guidelines of Revised National Tuberculosis Control Program in India (DOTS Category 1). The child responded well to the treatment within 8–10 weeks. On follow-up, there was a substantial reduction in the size of the swelling, restoration of the finger and toe movements, and healing of the sinus within 4-5 months.

## 3. Discussion

In childhood tuberculosis, the time between infection and development of symptomatic disease is very short. Without an adequate treatment, the risk of progression to active disease has been estimated to be higher in children (24% in 1–5 years of age) and increases again in adolescence (15%). Therefore, children are more prone to develop extrapulmonary tuberculosis [2].

The diagnostic delay in this child is attributed to

- Lack of high index of suspicion and poor awareness among the clinicians.
- Nonspecific clinical manifestations.
- Simultaneous involvement of both the limbs (usually bones of the hand are more commonly involved) [3].
- Presentation at an unusual age (uncommon beyond 6 years of age, once the epiphyseal centres are well established).
- Absence of concomitant pulmonary involvement.
- Paucibacillary nature of the lesion [4].

Therefore, a conglomeration of findings from careful history taking and physical examination supported with appropriate diagnostic work-up should form the basis of a prompt diagnosis of tuberculous dactylitis. As* Mycobacterium tuberculosis* does not produce proteolytic enzymes that can destroy the cartilage, there is potential for preservation of good function when early diagnosis is made [5]. Even in an endemic country like India where tuberculosis is rampant, the diagnosis is missed or often delayed as in this case, particularly due to usual absence of stigmata of pulmonary tuberculosis ending up with potentially fatal consequences. One must be vigilant while dealing with the pathology of short tubular bones of hands and feet, as various conditions like benign and malignant tumors, noninfectious granulomatous disease, sickle cell dactylitis, endocrinopathies, metabolic disorders, pyogenic and fungal osteomyelitis, Brodie's abscess, syphilitic dactylitis, brucellosis, and actinomycosis can mimic and resemble tuberculous dactylitis [6, 7].

However, it should be remembered that tuberculosis can present in the most unusual form and in least expected sites, so the budding clinicians are reminded of the importance of early diagnosis of this rare form of an ancient disease.

## References

[1] Salimpour R., Salimpour P. (1997). Picture of the month. Tuberculous dactylitis. *Archives of Pediatrics and Adolescent Medicine Journal*.

[2] Carrol E. D., Clark J. E., Cant A. J. (2001). Non-pulmonary tuberculosis. *Paediatric Respiratory Reviews*.

[3] Sunderamoorthy D., Gupta V., Bleetman A. (2001). TB or not TB: an unusual sore finger. *Emergency Medicine Journal*.

[4] Panchonia A., Kulkarni C. V., Meher R., Mandwariya S. (2012). Isolated tuberculous dactylitis [Spina ventosa] in a 9 year old boy—a rare entity. *International Journal of Basic and Applied Medical Sciences*.

[5] Nelson L. J., Wells C. D. (2004). Global epidemiology of childhood tuberculosis. *The International Journal of Tuberculosis and Lung Disease*.

[6] Maruschke L., Baumann T., Zajonc H., Herget G. (2013). Monostotic fibrous dysplasia of the middle phalanx of the hand. *Journal of Medical Cases*.

[7] Hassan F. O. A. (2010). Tuberculous dactylitis pseudotumor of an adult thumb: a case report. *Strategies in Trauma and Limb Reconstruction*.

